# Metabolomics Insights into the Differential Response of Breast Cancer Cells to the Phenolic Compounds Hydroxytyrosol and Luteolin

**DOI:** 10.3390/molecules28093886

**Published:** 2023-05-04

**Authors:** Maite Garcia-Guasch, Eduard Escrich, Raquel Moral, Iola F. Duarte

**Affiliations:** 1 Physiology Unit, Department of Cell Biology, Physiology and Immunology, Faculty of Medicine, Universitat Autònoma de Barcelona, 08193 Barcelona, Spain; mariateresa.garcia.guasch@uab.cat (M.G.-G.); eduard.escrich@uab.cat (E.E.); 2 CICECO—Aveiro Institute of Materials, Department of Chemistry, University of Aveiro, Campus de Santiago, 3810-193 Aveiro, Portugal

**Keywords:** breast cancer, metabolism, hydroxytyrosol, luteolin, EVOO phenolic compounds

## Abstract

The aim of this study was to investigate the effects of two phenolic compounds found in extra virgin olive oil, hydroxytyrosol (HT) and luteolin (LUT), on the metabolism of breast cancer (BC) cells of different molecular subtypes. An untargeted metabolomics approach was used to characterize the metabolic responses of both triple-negative MDA-MB-231 cells and hormone-responsive MCF-7 cells to treatment with these phenols. Notably, while some effects were common across both cell types, others were dependent on the cell type, highlighting the importance of cellular metabolic phenotype. Common effects included stimulation of mitochondrial metabolism, acetate production, and formate overflow. On the other hand, glucose metabolism and lactate production were differentially modulated. HT and LUT appeared to inhibit glycolysis and promote the hexosamine biosynthetic pathway in MDA-MB-231 cells, while MCF-7 cells exhibited higher glycolytic flux when treated with phenolic compounds. Another significant difference was observed in lipid metabolism. Treated MDA-MB-231 cells displayed increased levels of neutral lipids (likely stored in cytosolic droplets), whereas treatment of MCF-7 cells with HT led to a decrease in triacylglycerols. Additionally, glutathione levels increased in MDA-MB-231 cells treated with HT or LUT, as well as in MCF-7 cells treated with LUT. In contrast, in HT-treated MCF-7 cells, glutathione levels decreased, indicating different modulation of cellular redox status. Overall, this work provides new insights into the metabolic impact of HT and LUT on different BC cell subtypes, paving the way for a better understanding of the nutritional relevance of these phenolic compounds in the context of BC prevention and management.

## 1. Introduction

Breast cancer (BC) is the most common malignant tumour in women worldwide, displaying high incidence, prevalence, and mortality rates [1]. Among the several risk factors involved in BC pathogenesis, diet is an important environmental factor [2]. Higher adherence to the Mediterranean diet has been associated with a protective effect against several diseases, including BC [3]. The main source of lipids in the Mediterranean diet is olive oil, obtained from the fruit of the olive tree (*Olea europaea* L.). In addition to lipids, of which monounsaturated fatty acids (MUFAs) represent the largest fraction, extra virgin olive oil contains more than 230 components from different chemical classes, including triterpenes, sterols, hydrocarbons, squalene, n-alkanes and n-alkenes, carotenoids, lipophilic phenols, hydrophilic phenols, and others [4]. Many of these components have been related to cancer prevention due to their antitumor effects, especially hydroxytyrosol, secoiridoids, flavonoids, lignans, and triterpenes [5]. Hydroxytyrosol (HT) is a phenolic compound with a potential protective effect on BC, demonstrated both in vitro and in vivo. In vitro studies in several BC cells lines (MCF-7, MDA-MB-231, T47D, and SKBR3 cells) have shown HT to have antiproliferative and pro-apoptotic properties, along with the capacity to arrest the cell cycle and inhibit cell migration and invasion [6,7,8]. In Sprague Dawley rats with experimental mammary tumours, HT also inhibited cell proliferation and tumour growth by altering the expression of proliferation and apoptosis-related genes [9]. Luteolin (LUT) is another phenolic compound, of the flavonoid subclass, that has shown anti-proliferative properties, inhibition of cell growth, promotion of cell cycle arrest, induction of pro-apoptotic protein expression, and inhibition of cell migration and invasion in BC cell lines [10,11]. In vivo, LUT reduced tumour burden and growth in an MDA-MB-231 xenograft model [12,13]. It also inhibited tumour growth and angiogenesis in experimental mammary tumours [14].

In previous works, we have reported a potential protective effect of extra virgin olive oil (EVOO) on experimental mammary cancer. In the 7,12-dimethylbenz[a]anthracene (DMBA)-induced breast cancer model, animals fed a diet high in EVOO showed lower tumour incidence and yield, as well as a lower degree of morphological aggressiveness, in comparison to a high corn oil diet [14,15,16]. Subsequently, molecular analysis of experimental tumours revealed mechanistic differences, including in apoptosis, proliferation, and metabolism. In particular, the high-EVOO diet-induced altered expression of tumour proteins is known to be involved in several cell death pathways, suggesting a different balance in proliferation/apoptosis due to diet [17]. Moreover, animals fed the EVOO-rich diet showed modified glucose and mitochondrial metabolism when compared to animals fed the corn oil-enriched diet [18]. Interestingly, in vitro assays carried out in different human BC cell lines showed EVOO minor compounds (especially HT), and not fatty acids, to be largely responsible for the effects observed in vivo [18]. Hence, in the present work, we aimed to further dissect the impact of two EVOO phenols, HT and LUT, on BC cell metabolism.

Metabolic reprogramming is an important cancer hallmark, involved in many aspects of tumour initiation and progression [19]. The most emblematic oncogenesis-related metabolic alteration, known as the Warburg effect, consists of upregulated glycolytic activity and lactate production, even in aerobic conditions, to support increased energetic and biosynthetic needs [20,21]. Moreover, cancer cells typically present altered amino acid metabolism (such as increased glutaminolysis) and upregulation of one-carbon metabolism, together with increased lipid uptake, lipogenesis, and cholesterol synthesis [22]. Metabolic profiling of in vitro cultured cells, mainly using NMR- or MS-based metabolomics, represents an exquisitely valuable tool to study cell metabolism and its modulation with exogenous compounds, such as anti-cancer phytochemicals [23,24]. Previous studies have used metabolomics to investigate the metabolic responses of BC cells to polyphenols such as curcumin [25], soy isoflavones [26], and a panel of five compounds (quercetin, curcumin, tannic acid, genistein, and resveratrol) [27]. However, to the best of our knowledge, the metabolic effects of HT and LUT, two important constituents of extra virgin olive oil, on BC cells have been poorly studied.

In this work, an untargeted metabolomics approach based on ^1^H NMR spectroscopy of cell extracts and culture medium supernatants was used to characterize the changes in BC cell metabolism induced with HT and LUT. Two cell lines, which represent distinct BC molecular subtypes, were used, namely the highly aggressive triple-negative (TNBC) MDA-MB-231 cells and the luminal A-like, hormone-responsive MCF-7 cells. The results are expected to contribute to an improved understanding of the role of nutritional factors on BC metabolism and pathogenesis.

## 2. Results

### 2.1. Cell Viability

**Figure 1:**
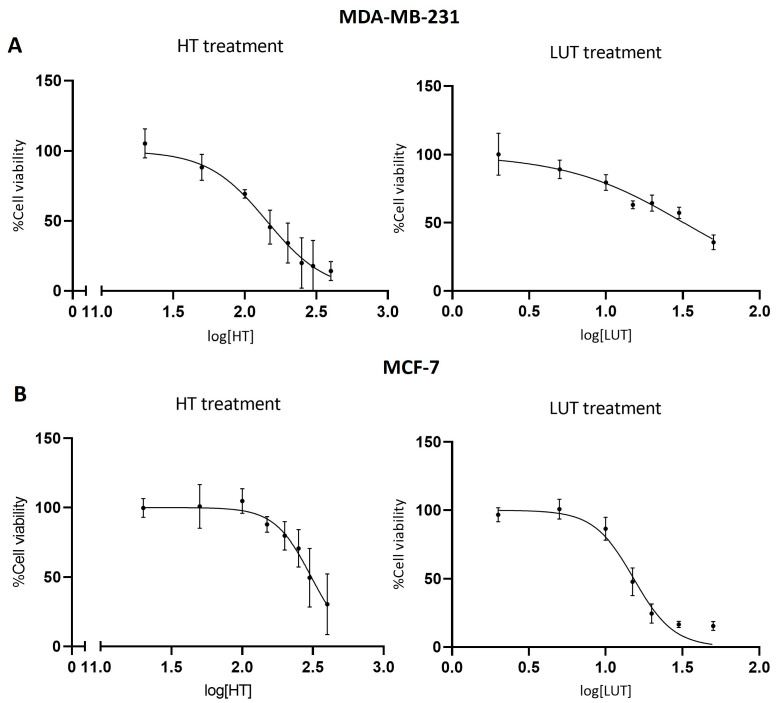
Viability of breast cancer cells upon 72 h treatment with different concentrations of HT or LUT, as assessed with an MTT assay. (**A**) Viability of MDA-MB-231 cells treated with HT (left) or LUT (right). (**B**) Viability of MCF-7 cells treated with HT (left) or LUT (right).

The two phenolic compounds differentially affected the viability of the cell lines tested, as shown by the MTT results (Figure 1). In both cell lines, LUT decreased cell viability at lower concentrations than HT. For HT, the concentrations causing a 50% decrease in cell viability (IC50) were 142 μM in MDA-MB-231 cells and 308 μM in MCF-7 cells. For LUT, the IC50 values were 32 μM in MDA-MB-231 cells and 15 μM in MCF-7 cells. Thus, MDA-MB-231 were affected by HT at lower concentrations, whereas MCF-7 cells were more sensitive to LUT treatment. Based on these results, IC50 concentrations and their half values were selected for metabolomics studies.

### 2.2. Extracellular Metabolic Profiling

**Figure 2:**
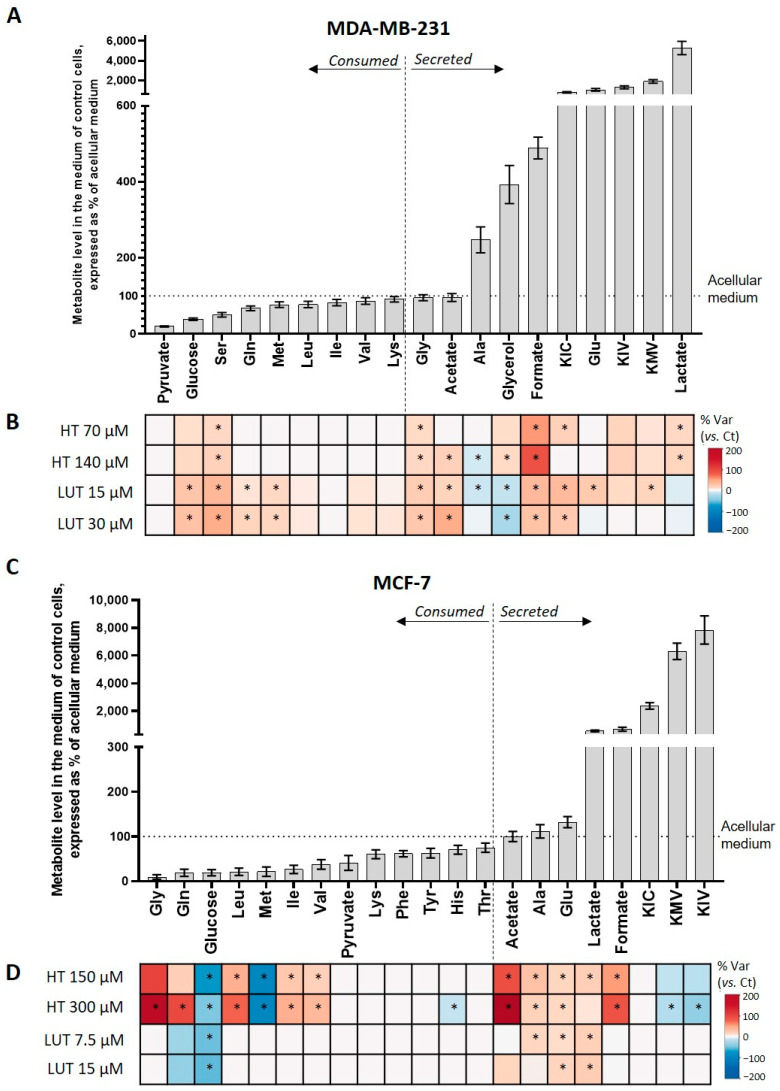
Metabolic profiling of culture medium. (**A**) Metabolites consumed and secreted by untreated MDA-MB-231 cells during 72 h in culture; the variations are expressed in relation to metabolite levels in an acellular medium (set to 100%, dashed line). (**B**) Heatmap coloured according to the % of the variation in MDA-MB-231 cells treated with HT (70 µM and 140 µM) or LUT (15 µM and 30 µM) in relation to untreated controls. (**C**) Metabolites consumed and secreted by untreated MCF-7 cells during 72 h in culture; the variations are expressed in relation to metabolite levels in an acellular medium (set to 100%, dashed line). (**D**) Heatmap coloured according to the % of the variation in MCF-7 cells treated with HT (150 µM and 300 µM) and LUT (7.5 µM and 15 µM) in relation to untreated controls. For metabolites consumed, the red colour represents higher levels in the medium, hence, lower consumption, while the blue colour represents lower levels in the medium, hence, increased consumption. For metabolites secreted, red and blue colours represent, respectively, higher and lower secretions. The colour intensity reflects the magnitude of consumption/excretion. Statistical significance was assessed with respect to controls using the *t*-test (* *p* < 0.05).

For each cell type, ^1^H NMR profiling of culture medium supernatants revealed the impact of treatment with the phenolic compounds on the cells’ consumption and secretion patterns. Non-treated MDA-MB-231 cells used up pyruvate (80% consumed), glucose (61% consumed), and several amino acids (Figure 2A), among which serine was the most extensively consumed (50%), followed by glutamine (32%), methionine (23%), and BCAA (15–22%). Concomitantly, MDA-MB-231 cells secreted high amounts of lactate, alanine, glutamate, formate, glycerol, and branched-chain keto acids (BCKAs), namely α-ketoisocaproate (KIC), α-ketomethylvalerate (KMV), and α-ketoisovalerate (KIV). Treatment with HT mainly affected the consumption of serine (decreased in comparison to untreated controls) and the secretion of alanine (decreased), lactate, formate, and glycerol (increased) (Figure 2B and Supplementary Table S1). Moreover, glycine and acetate were newly secreted by HT-treated MDA-MB-231 cells. In regard to LUT, it induced MDA-MB-231 cells to decrease the consumption of glucose and some amino acids, as well as the secretion of alanine and glycerol. On the other hand, the release of acetate, formate, glycine, BCKA, and glutamate (at lower exposure concentrations) increased in LUT-treated MDA-MB-231 cells.

As for MCF-7 cells, their preferred substrates were glucose (81%), pyruvate (59%), and several amino acids (Figure 2C). Among those, glycine and glutamine were the most extensively consumed (91% and 81%) followed by methionine (79%) and the branched-chain amino acids (BCAAs) leucine (79%), isoleucine (74%), and valine (63%). On the other hand, BCKAs were secreted along with formate, lactate, glutamate, and alanine. Upon treatment with phenols, MCF-7 cells displayed several changes in extracellular metabolite levels (Figure 2D and Supplementary Table S2). In the presence of HT, they significantly increased the consumption of glucose, methionine, and histidine (these metabolites showed lower levels in the medium of treated vs. control cells), while using up less glycine, glutamine, and BCAAs (higher levels in treated vs. control media). In terms of secreted metabolites, HT-treated MCF-7 cells newly secreted acetate and increased the release of lactate, alanine, glutamate, and formate, whereas the secretion of KMV and KIV was reduced. Although less extensive than HT, LUT also altered the MCF-7 cells’ extracellular profile (Figure 2D). Specifically, compared to untreated controls, the main changes were increased glucose consumption and increased secretion of lactate, alanine, and glutamate.

Overall, the two phenolic compounds significantly and differentially affected the exometabolome of MDA-MB-231 and MCF-7 cells. Interestingly, the metabolic activity of MDA-MB-231 cells appeared to be more extensively modulated by LUT, whereas HT had a greater impact on MCF-7 cells’ exometabolome. Furthermore, the observed effects were highly dependent on the cell line. For instance, both phenols, especially LUT, decreased glucose consumption in MDA-MB-231 cells, whereas treated MCF-7 cells consumed more glucose and secreted more lactate, likely indicating a different impact on glycolysis, as discussed next.

### 2.3. Intracellular Metabolic Profiling

**Figure 3:**
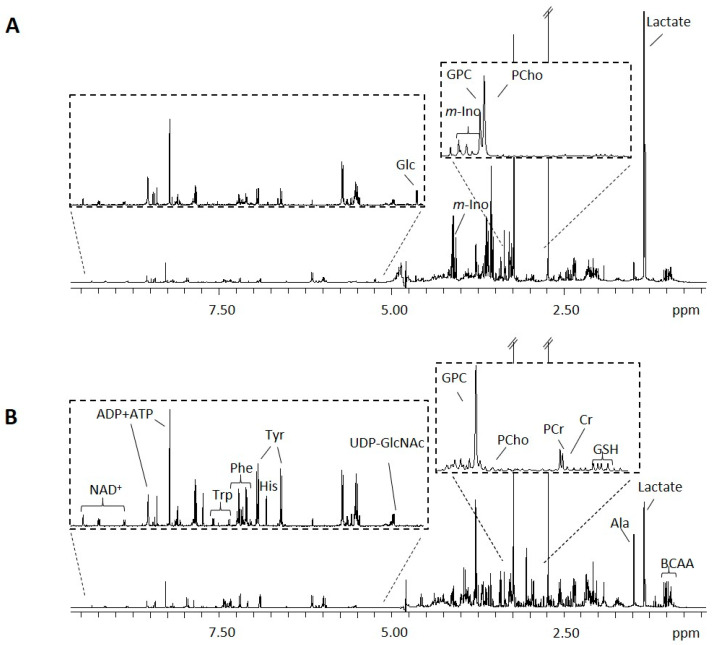
The 1D ^1^H NMR spectra of polar extracts from (**A**) MDA-MB-231 cells and (**B**) MCF-7 cells. Three-letter codes used for amino acids; ATP/ADP, adenosine tri/di-phosphate; BCAAs, branched-chain amino acids; Cr, creatine; Glc, glucose; GPC, glycerophosphocholine; GSH, reduced glutathione; NAD^+^, nicotinamide adenine dinucleotide; PCho, phosphocholine; PCr, phosphocreatine; UDP-GlcNAc, uridine diphosphate N-acetylglucosamine.

The first important observation is that the two cell lines presented markedly different intracellular profiles (Figure 3 and Supplementary Figure S1A). For instance, the intracellular levels of most amino acids (e.g., alanine, lysine, arginine, BCAAs, aromatic amino acids), creatine, phosphocreatine, and glutathione were significantly higher in MCF-7 than in MDA-MB-231 cells, while the latter presented increased relative abundances of lactate, PCho, *myo*-inositol, proline, and glycine.

**Figure 4:**
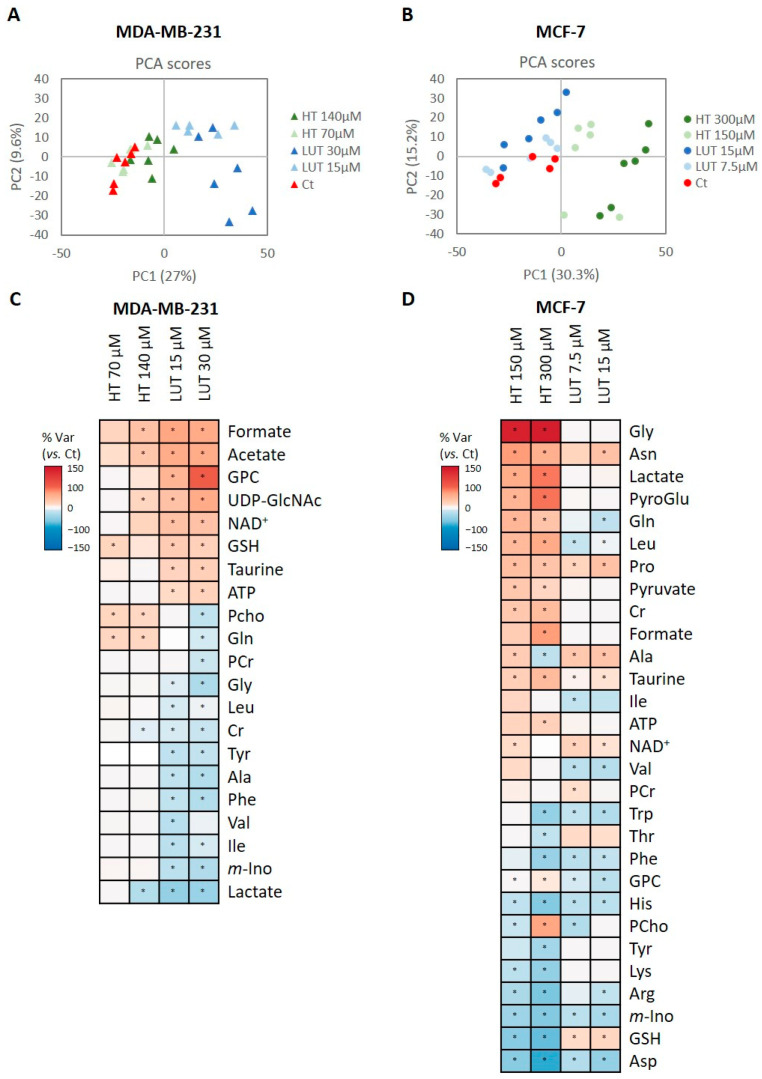
Metabolic profiling of cell polar extracts. (**A**,**B**) Score scatter plots obtained with a principal component analysis (PCA) of the spectral profiles obtained for MDA-MB-231 and MCF-7 cells, respectively. (**C**,**D**) Heatmaps of the metabolic variations induced by HT and LUT on intracellular metabolite levels of MDA-MB-231 and MCF-7 cells, respectively. The colour scale represents the % of the variation in relation to control cells (positive variations—red scale—for increased metabolites, and negative variations—blue scale—for decreased metabolites). Statistical significance was assessed with respect to controls using the *t*-test (* *p* < 0.05). Three-letter codes used for amino acids; ATP, adenosine triphosphate; Cho, choline; Cr, creatine; GPC, glycerophosphocholine; GSH, reduced glutathione; *m*-Ino, *myo*-inositol; NAD^+^, nicotinamide adenine dinucleotide; PCho, phosphocholine; PCr, phosphocreatine; UDP-GlcNAc, uridine diphosphate N-acetylglucosamine.

The influence of the two phenolic compounds on the intracellular metabolic composition of MDA-MB-231 and MCF-7 cells was first assessed with multivariate analysis of the ^1^H NMR spectra recorded for cell polar extracts. In MDA-MB-231 cells, a principal component analysis (PCA) revealed a clear separation along PC1 (27% explained variance) between untreated controls (CTs) and the cells treated with LUT (Figure 4A). The separation between CT and HT-treated cells was less clear when the five groups were considered together; hence, each treatment was also analysed separately. The results, shown in Supplementary Figure S2A, indicate a dose-dependent separation from the CT for both phenols. A partial least squares discriminant analysis (PLS-DA) confirmed this separation to be more robust for LUT than for HT (Q^2^ 0.669 vs. Q^2^ 0.478), reflecting the higher metabolic impact of LUT on the intracellular composition of MDA-MB-231 cells. The corresponding LV1 loading profiles (Supplementary Figure S2B) further revealed the main metabolites that varied in response to HT or LUT, corroborating the stronger impact of LUT and highlighting some differential effects between the two phenolic compounds.

Contrarily to MDA-MB-231 cells, in the case of MCF-7 cells, the PCA separation of sample groups was clearer for HT-treated samples (Figure 4B). This result was validated with PLS-DA (Supplementary Figure S3A), as HT-treated cells were discriminated from CT samples with higher predictive power than LUT-treated cells (Q^2^ 0.572 vs. Q^2^ 0.356). Accordingly, the corresponding LV1 loading profiles (Supplementary Figure S3B) explaining HT effects were more intensely coloured than those relating to LUT effects.

To evaluate the magnitude of HT and LUT intracellular metabolic effects in more detail, the levels of individual metabolites (assessed with spectral integration) in the treated cells were measured in relation to their respective controls. The results are summarized as heatmaps in Figure 4C,D for MDA-MB-231 and MCF-7 cells, respectively, while numerical variations are presented in Supplementary Tables S3 and S4. Regarding MDA-MB-231 cells (Figure 4C), only eight intracellular metabolites were significantly altered in response to HT, namely formate, acetate, uridine diphosphate-N-acetylglucosamine (UDP-GlcNAc), glutathione (GSH), phosphocholine (PCho), and glutamine (increased) together with creatine (Cr) and lactate (decreased). As seen for the exometabolome, LUT had a stronger effect on the intracellular composition of this cell line, significantly affecting the levels of twenty-one metabolites. Formate, acetate, UDP-GlcNAc, glycerophosphocholine (GPC), GSH, taurine, NAD^+^, and ATP were increased, whereas lactate, PCho, *myo*-inositol, Cr, phosphocreatine (PCr) and several amino acids were decreased relative to control cells.

Regarding MCF-7 cells (Figure 4D), twenty-nine metabolites showed significantly different levels (*p* < 0.05) in HT-treated cells compared to untreated controls, while nineteen varied significantly due to LUT exposure. In particular, HT treatment increased the intracellular levels of lactate, pyruvate, formate, Cr, taurine, a number of amino acids (asparagine, glycine, glutamine, pyroglutamate, leucine, and proline), NAD^+^, and ATP while decreasing the levels of another set of amino acids (aspartate, arginine, lysine, threonine, aromatic amino acids, and histidine), GSH, and *myo*-inositol. Most of these changes increased in magnitude with HT concentration, whereas Ala, GPC, and PCho showed opposite variations at low and high HT doses. As for LUT-treated cells, the main variations compared to controls comprised increases in the levels of asparagine, proline, alanine, taurine, PCr, NAD^+^, and GSH, together with decreased levels of GPC, *myo*-inositol and amino acids such as aspartate, glutamine, BCAAs, and aromatic amino acids.

### 2.4. Lipid Composition

The ^1^H NMR analysis of the cells’ non-polar extracts allowed several lipid classes to be identified and their relative levels in the two cell lines to be compared. While the contents of total fatty acids (FAs) and phosphatidylcholine (PC) were similar, there were significant differences in the abundance of cholesterol (higher in MDA-MB-231), triacylglycerols (TAG), and phosphatidylethanolamine (PE) (higher in MCF-7), as shown in Supplementary Figure S1B. Moreover, MDA-MB-231 cells showed lower levels of unsaturated FAs (UFAs) but a higher relative proportion of polyunsaturated FAs (PUFAs).

**Figure 5:**
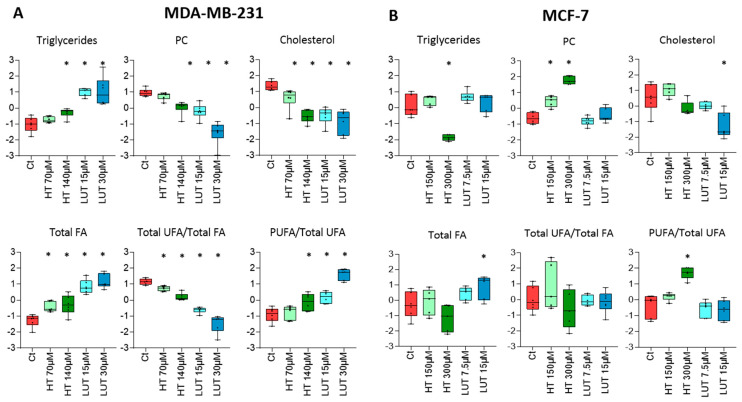
Metabolic profiling of cell non-polar extracts. (**A**,**B**) Relative levels of different lipid classes in MDA-MB-231 and MCF-7 cells, respectively. Signal integrals were mean-centred and scaled to unit variance. Statistical significance was assessed with respect to controls using ANOVA (* *p* < 0.05). FAs, fatty acids; PC, phosphatidylcholine; PUFAs, polyunsaturated fatty acids; UFAs, unsaturated fatty acids.

Regarding the variations in treated vs. control cells, in MDA-MB-231 cells (Figure 5A), HT and LUT caused an increase in TAG and total FA, accompanied with decreases in PC and cholesterol. These changes were more prominent for LUT than for HT. Regarding the FA composition of these lipids, total UFAs decreased in treated cells, but the relative contribution of PUFAs to the pool of unsaturated FAs increased. In MCF-7 cells (Figure 5B), HT treatment induced a decrease in TAG and an increase in PC, together with an increase in the PUFA/UFA ratio, whereas LUT did not change these lipids. On the other hand, only LUT decreased cholesterol levels.

## 3. Discussion

In this work, we assessed the metabolic impact of two phenolic constituents of EVOO (HT and LUT) on BC cell lines representing different molecular subtypes and clinical features, namely, the highly invasive triple-negative MDA-MB-231 cells and the less aggressive luminal A-like MCF-7 cells.

The cell viability results showed that both phenolic compounds were cytotoxic to the tested BC cell lines, although to different extents. In particular, HT was found to affect cell viability at higher concentrations than LUT, implying a higher cytotoxicity for the latter. Moreover, the two cell lines were differentially sensitive to the phenols. In agreement with previous studies [7,28], HT was more cytotoxic to MDA-MB-231 than to MCF-7 cells (IC50 140 vs. 300 µM). On the other hand, LUT displayed higher cytotoxicity in MCF-7 than in MDA-MB-231 cells (IC50 15 vs. 30 µM), which is also in line with previous findings [10,11].

**Figure 6:**
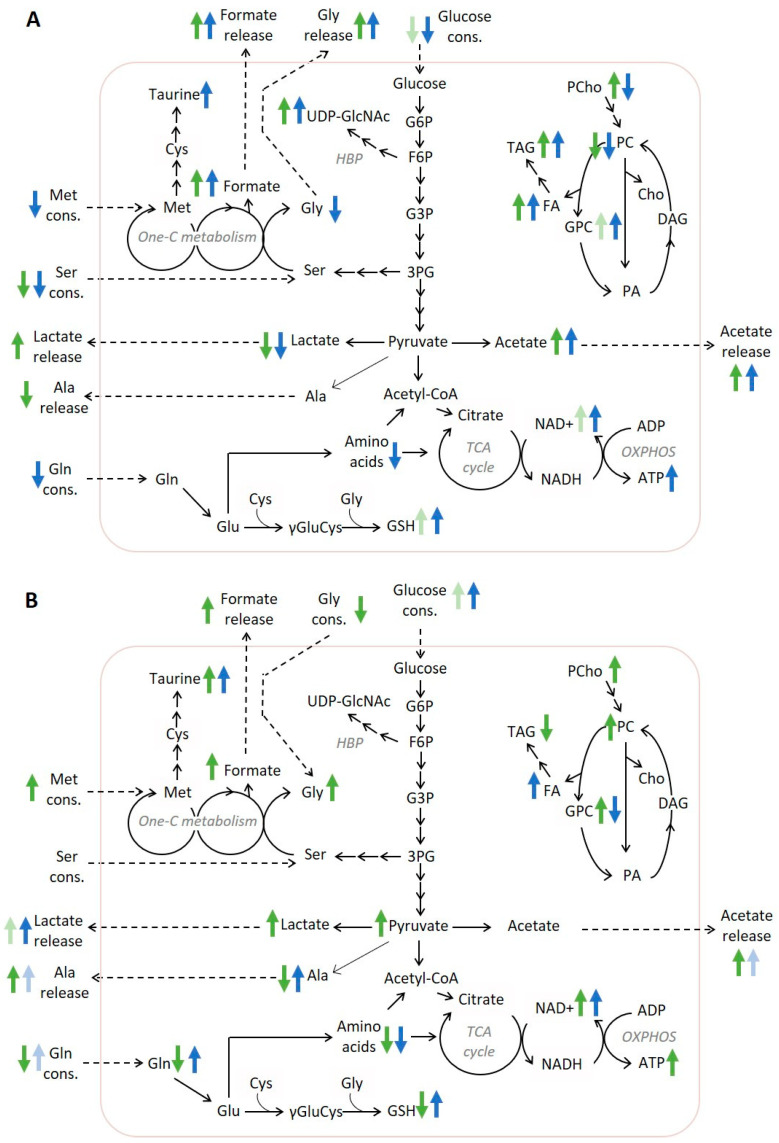
Schematic diagram showing the main metabolic responses of (**A**) MDA-MB-231 and (**B**) MCF-7 cells to HT (green arrows) and LUT (blue arrows) at the respective IC50 concentrations. Changes that did not reach statistical significance in relation to controls (*p* > 0.05) are represented with lighter arrows.

At the metabolic level, the two cell types also responded differently to treatment, as revealed with the integrated exo- and endometabolomics approach used herein. The main findings are summarized in Figure 6A,B for MDA-MB-231 and MCF-7 cells, respectively, and discussed below.

In MDA-MB-231 cells, treatment with phenolic compounds decreased glucose consumption and intracellular lactate levels, which, in the case of LUT, was also accompanied by decreased lactate secretion, suggesting downregulation of glucose uptake and/or glycolytic turnover. These results agree with previous studies associating the anti-neoplastic activity of phenols with their ability to interfere with the high reliance of cancer cells on glycolysis (Warburg effect) [29,30]. One of the main mechanisms reported to be involved is the inhibition of glucose uptake, mainly through decreased expression of the facilitative glucose transporter GLUT1. Indeed, LUT has been recently highlighted as one of the phenolic compounds with the strongest GLUT1 inhibitory activity in human embryonic kidney cells (HEK293T) that present stable expression of this receptor [31]. Moreover, some phenols such as curcumin, oroxylin A, and resveratrol were reported to directly interfere with glycolytic enzymes and glycolytic flux [29]. In the case of LUT (50 and 100 µM), a previous work has shown it to decrease lactate production in MCF-7 and 4T1 BC cells, but only when they were incubated under hypoxia, having no impact on the glycolytic flux under normoxic conditions [32]. As for HT, to our knowledge, its impact on glucose transport and metabolism has not been previously addressed in the literature. The intracellular accumulation of UDP-GlcNAc observed herein for treated MDA-MB-231 cells may also relate to glucose metabolism reprogramming, as it indicates the diversion of the glycolytic intermediate fructose-6-phosphate into the hexosamine biosynthetic pathway (HBP). Indeed, UDP-GlcNAc is the end product of HBP and a key substrate for the glycosylation of proteins that regulate nutrient sensing and stress response [33].

Interestingly, the impact of the phenolic compounds on glucose metabolism was markedly different in MCF-7 cells compared to MDA-MB-231 cells. MCF-7 cells displayed higher glucose consumption, lactate secretion, and higher intracellular lactate levels (in the case of HT-treated cells), indicating an increase in glycolytic flux. According to our own results and the data of others [34,35,36], in basal conditions, MCF-7 cells tend to be less glycolytic than MDA-MB-231 cells, which may partially justify their differential regulation of glycolysis upon treatment with the phenols. A few other studies have also reported phenolic compounds such as Gossypol or Catechin to stimulate glucose uptake by MCF-7 cells [37,38], which is a response that may be viewed as an adaptive mechanism to oxidative stress [39].

Our results further indicate that both cell types upheld or intensified mitochondrial metabolism upon treatment with phenols. Indeed, when treated with HT or LUT, MDA-MB-231 and MCF-7 cells maintained extensive consumption of extracellular pyruvate and displayed unchanged or increased intracellular levels of ATP and NAD^+^ (consumed in glycolysis and regenerated in oxidative phosphorylation—OXPHOS). Moreover, several intracellular amino acids were significantly depleted, possibly reflecting their use as anaplerotic substrates to fuel the TCA cycle. Despite several phenolic compounds being reported to interfere with electron transport chain complexes and act as mitochondrial uncouplers [40], a boost in mitochondrial respiration has also been observed in a few studies. For instance, in colon cancer cells, resveratrol downregulated glycolysis and stimulated OXPHOS, leading to increased ATP production [41,42]. A similar effect was reported in MDA-MB-231 cells treated with a eucalyptus bark extract [43]. Moreover, HT was recently reported to enhance the protein levels of respiratory chain complexes and ATP production in colorectal cancer cells [44].

Another interesting observation, common to the two cell lines, was the increase in acetate (intracellular and/or extracellular) in both HT- and LUT-treated cells, especially at the higher exposure concentrations. According to the seminal work by Liu et al. [45], acetate overflow may result from pyruvate through its non-enzymatic, ROS-mediated conversion or from the altered activity of keto acid dehydrogenases. In the context of the present work, it is plausible to speculate that polyphenol-induced ROS may be involved, although this hypothesis requires further investigation.

MDA-MB-231 cells treated with either HT or LUT and MCF-7 cells treated with HT additionally exhibited enhanced release and intracellular levels of formate. Based on the knowledge that formate overflow in cancer cells mainly results from serine catabolism in excess of one-carbon unit required for biosynthesis [46], this observation is consistent with decreased cell proliferation and, hence, reduced biosynthetic needs. Concordantly, MDA-MB-231 cells treated with these phenolic compounds consumed less serine and glutamine while secreting more glycine and formate. In the case of MCF-7 cells, glycine switched from being released to being consumed, likely due to the lower serine levels available in the medium of these cells [46]. Consistent with lower proliferation, the consumption of glycine, as well as glutamine and BCAAs, decreased in HT-treated MCF-7 cells, while LUT had no significant impact. Interestingly, HT-treated MCF-7 cells significantly increased the consumption of methionine. This essential amino acid serves many important roles in cancer cells, such as participating in methylation reactions, contributing to the folate cycle (hence, one-carbon metabolism), fuelling the synthesis of polyamines, purines, and pyrimidines, and contributing to redox regulation by providing homocysteine as a substrate for the transsulfuration pathway involved in the synthesis of GSH [47]. Its specific role in the response of MCF-7 cells to HT warrants further investigation.

GSH represents a key endogenous antioxidant defence, with a crucial role in suppressing ROS and supporting tumour cell survival, namely in TNBC [48]. In the present work, GSH levels increased upon the treatment of MDA-MB-231 cells with HT or LUT and in MCF-7 cells exposed to LUT. These results agree with other studies reporting increased GSH synthesis and/or regeneration upon treatment of cancer and non-cancer cells with HT [49,50] or LUT [51]. Indeed, phenolic compounds are widely recognized for their ability to modulate oxidative stress in cancer cells, exhibiting either antioxidant or pro-oxidant activities depending on the conditions [52]. Notably, in HT-treated MCF-7 cells, GSH levels decreased compared to untreated controls, while its precursor amino acids glycine and glutamine were increased, suggesting downregulated GSH synthesis.

The analysis of cellular non-polar extracts provided additional insights into lipid metabolism. Treatment with HT or LUT resulted in increased levels of TAG and total FAs, as well as a decrease in PC content in MDA-MB-231 cells, with these changes being more pronounced for LUT. Moreover, LUT-treated cells exhibited accumulation of GPC and decreased PCho (detected in the polar extracts), together with lower glycerol release. Altogether, these variations suggest the breakdown of membrane glycerophospholipids and the incorporation of their fatty acids into neutral lipids (stored in cytosolic droplets). The build-up of lipid droplets (LDs) is a common adaptive response to cellular stress caused by redox imbalance, nutrient starvation, and other factors [53]. On the other hand, in MCF-7 cells, HT triggered the strongest changes in TAG and PC levels, and the results suggested degradation of lipid droplets in contrast to the response observed in MDA-MB-231 cells. Treatment with phenolic compounds further induced decreased cholesterol levels in both cell lines and changes in the proportion of unsaturated fatty acids, corroborating the impact on cell membrane composition.

## 4. Materials and Methods

### 4.1. Chemicals

Hydroxytyrosol (PHL80152), luteolin (L9283), dimethylsulfoxide (DMSO), methanol, chloroform, deuterium oxide (D_2_O) containing 0.75% 3-(trimethylsilyl) propionic acid (TSP-d4), and MTT (3-(4,5-Dimethyl-2-thiazolyl)-2,5-diphenyl-2H-tetrazolium bromide, thiazole blue) were purchased from Sigma-Aldrich (Darmstadt, Germany). Gibco^TM^ Dulbecco’s Modified Eagle Medium (DMEM), Gibco^TM^ Eagle’s Minimum Essential Medium (EMEM), Gibco^TM^ Fetal Bovine Serum (FBS), insulin, and phosphate buffer saline (PBS) were obtained from Thermo Fisher Scientific (Waltham, MA, USA). Deuterium oxide 100% and deuterated chloroform containing 0.03% tetramethylsilane (TMS) were purchased from Cortecnet (Paris-Saclay, France).

### 4.2. Instrumentation

NMR spectra were acquired using a Bruker Avance III HD 500 NMR spectrometer (University of Aveiro, Portuguese NMR Network) operating at 500.13 MHz for ^1^H observation and equipped with a 5 mm TXI probe.

### 4.3. Cell Culture

The human breast cancer cell lines MDA-MB-231 and MCF-7 were obtained from the American Type Culture Collection (ATCC, LGC Standards, Middlesex, UK). MDA-MB-231 cells were grown in DMEM supplemented with 10% FBS. MCF-7 cells were grown in EMEM, supplemented with 10% foetal bovine serum (FBS) and 0.01 mg/mL of insulin. The cells were maintained at 37 °C in a humidified atmosphere of 95% air and 5% CO_2_.

### 4.4. Viability Assay

Cell viability was determined with the colorimetric MTT assay, which measures the intracellular reduction of tetrazolium salts into purple formazan by viable cells [54]. MDA-MB-231 and MCF-7 cells were treated with HT at 0, 20, 50, 100, 150, 200, 250, 300, and 400 µM or with LUT at 0, 2, 5, 10, 15, 20, 30, and 50 µM for 72 h. All the solutions contained 0.2% of DMSO. Cells were seeded into 96-well plates at a density of 5 × 10^3^ cells/well for MDA-MB-231 and 6 × 10^3^ cells/well for MCF-7 and incubated overnight. The cell culture medium was replaced with fresh media containing HT or LUT and incubated for 72 h. The MTT reagent was added to each well (10 µL/well of MTT solution at 5 mg/mL in PBS) and incubated for 2 h at 37 °C. Then, formazan crystals were solubilized in 100 µL of 100% DMSO and absorbances were read at 570 nm. Relative cell viability was calculated as the absorbance ratio of treated and control samples.

### 4.5. Cell Treatment for NMR Analysis

MDA-MB-231 and MCF-7 cells were seeded into 100 mm cell culture dishes at a density of 2 × 10^5^ cells/mL with a complete medium and allowed to recover for 48 h. Then, the cell culture medium was replaced with media containing the phenolic compounds and incubated for 72 h. For treatments, MDA-MB-231 cells were incubated with HT at 0, 70, and 140 µM or LUT at 0, 15, and 30 µM. MCF-7 cells were incubated with HT at 0, 150, and 300 µM or LUT at 0, 7.5, and 15 µM. For all treatments, a culture medium without cells was placed under the same conditions. Three independent assays with duplicates were performed, giving a total of six replicate samples per condition and per cell type.

### 4.6. Sample Collection and Preparation for NMR Analysis

Medium samples and cell extracts were prepared as previously described [36]. Briefly, to precipitate medium proteins and retain the metabolites, cold methanol was added to medium samples (2:1 volume ratio), and the supernatant was recovered after 30 min at −20 °C followed by centrifugation (13,000× *g*, 20 min, 4 °C). After PBS washing, cells were recovered from the culture dishes by scraping in cold methanol, followed by the addition of chloroform and water (final volume ratios 1:1:0.7) and centrifugation (10,000× *g*, 15 min, 4 °C) to separate the polar and non-polar phases. Polar extracts and medium supernatants were then dried in a Speedvac concentrator (Thermo Fischer Scientific), while non-polar extracts were dried under a nitrogen stream. All samples were stored at −80 °C. For NMR analysis, medium samples and polar extracts were reconstituted in 600 µL of deuterated phosphate buffer (PBS 100 mM, pH 7.4) containing 0.1 mM TSP-d4, and the organic phases were reconstituted in 600 µL deuterated chloroform containing 0.03% TMS. The samples (550 μL) were then transferred into 5 mm NMR tubes for analysis.

### 4.7. NMR Data Acquisition

Standard 1D ^1^H spectra (Bruker pulse programs “noesypr1d” for aqueous extracts and “zg” for organic extracts) were recorded with a 7002.8 Hz spectral width, 32 k data points, a 2 s relaxation delay, and 512 scans. In addition, 2D ^1^H–^1^H total correlation spectra (TOCSY) and J-resolved spectra were also registered for selected samples to assist spectral assignment. Spectral processing was performed using Topspin 4.0.6 (Bruker BioSpin, Rheinstetten, Germany) and comprised manual phasing, baseline correction, and signal calibration with the TSP/TMS signal (0 ppm). Signal assignment was based on matching 1D and 2D spectral information to reference spectra available in the Bruker database BBiorefcode, as well as in Chenomx 9.0 (Edmonton, AB, Canada) and the HMDB Compound Reference Library [55].

### 4.8. Multivariate Analysis and Spectral Integration of NMR Spectra

Spectra were normalized by total area and data matrices were scaled to unit variance (UV) giving equal variance to all variables. Principal component analysis (PCA) and partial least squares discriminant analysis (PLS-DA) were applied to data using SIMCA-P 11.5 (Umetrics, Umeå, Sweden). The results were represented as score scatter plots, and the cross-validated explained variance (Q^2^) was used to assess the robustness of the PLS-DA group discrimination.

To provide a quantitative measurement of metabolic variations, selected signals were integrated using Amix-Viewer 3.9.15 (Bruker Biospin) and normalized by total area. For each metabolite, the percentage of variation in the treated samples was calculated relative to controls, along with the effect size (ES) and statistical significance (*p*-value). Loadings profiles and heatmap figures were generated using the R software version 4.1.3 (R Core Team (2020). R: A language and environment for statistical computing. R Foundation for Statistical Computing, Vienna, Austria. URL http://www.R-project.org/) (accessed on 7 March 2023).

## 5. Study Limitations

This study was conducted using BC cell lines, which are simplified models that cannot completely replicate tumour heterogeneity or the complex cellular interactions and microenvironmental factors present in vivo. Another important limitation is that metabolomics provides a static snapshot of the metabolites present in the sample, which may not accurately reflect the dynamic changes that occur in vivo. Additionally, metabolomics does not provide information on the mechanisms that regulate metabolic pathways or the factors that drive metabolic reprogramming. Hence, future work should integrate metabolomics with other omics approaches, such as transcriptomics or proteomics, to provide more comprehensive insights into the underlying biological mechanisms for metabolic changes.

## 6. Conclusions

Integrated exo-and endometabolomics of BC cells cultured in vitro revealed extensive metabolic reprogramming upon treatment with the phenolic compounds HT and LUT at their respective IC50 concentrations. In general, the metabolome of MDA-MB-231 cells was more significantly affected by LUT, whereas HT had a greater impact on the metabolic profile of MCF-7 cells. Furthermore, although some effects were shared between the two cell lines, others were markedly distinct, underscoring the significance of metabolic context in cancer metabolomics studies. Common effects included stimulation of mitochondrial metabolism (with NAD^+^ and ATP levels being increased or unaltered), acetate production (possibly from ROS-induced conversion of pyruvate) and formate overflow (likely reflecting one-carbon metabolism adaptation to lower biosynthetic needs). On the other hand, the modulation of glycolysis and lipid metabolism was clearly cell-type-dependent. In MDA-MB-231 cells, the phenol treatments appeared to inhibit glycolysis (promoting HBP) and stimulate the build-up of neutral lipids. In contrast, the results for treated MCF-7 cells suggested an upregulation in the glycolytic flux and, in the case of HT exposure, the breakdown of neutral lipids. Furthermore, in MCF-7 cells, HT induced specific effects such as upregulated methionine consumption and decreased intracellular levels of GSH, possibly indicating higher oxidative stress. Overall, the results of this study provide novel clues about the metabolic impact of HT and LUT on different BC cell subtypes, which can contribute to a better understanding of the nutritional relevance of these phenolic compounds in the context of BC prevention and management.

## Data Availability

The data supporting the reported results are available from the corresponding authors upon reasonable request.

## Supplementary Materials

The following supporting information can be downloaded at: https://www.mdpi.com/article/10.3390/molecules28093886/s1, Figure S1. Heatmaps illustrating the levels of (A) intracellular polar metabolites and (B) lipids in MDA-MB-231 and in MCF-7 cells. Figure S2. Multivariate analysis of the spectral profiles from MDA-MB-231 cell polar extracts. Figure S3. Multivariate analysis of the spectral profiles from MCF-7 cell polar extracts. Table S1. Metabolite variations in the culture medium of MDA-MB-231 cells treated with HT or LUT in relation to untreated controls. Table S2. Metabolite variations in the culture medium of MCF-7 cells treated with HT or LUT in relation to untreated controls. Table S3. Metabolite variations in the polar extracts of MDA-MB-231 cells treated with HT or LUT in relation to untreated controls. Table S4. Metabolite variations in the polar extracts of MCF-7 cells treated with HT or LUT in relation to untreated controls.

## Sample Availability

Samples of the compounds are not available from the authors.
