# Supplementary material for: Metabolomics Insights into the Differential Response of Breast Cancer Cells to the Phenolic Compounds Hydroxytyrosol and Luteolin
Source: Molecules. 2023 May 4;28(9):3886. doi: 10.3390/molecules28093886 (PMC10179918; doi:10.3390/molecules28093886)
Supplement: Supplementary file 1 [file molecules-28-03886-s001.zip › molecules-2374920-supplementary.pdf]

Supplementary Material

Metabolomics Insights into the Differential Response of Breast Cancer Cells to the Phenolic Compounds Hydroxytyrosol and Luteolin

Maite Garcia-Guasch [1], Eduard Escrich [1], Raquel Moral [1,*] and Iola F. Duarte [2,*]

[1] Physiology Unit, Department of Cell Biology, Physiology and Immunology, Faculty of Medicine,
Universitat Autònoma de Barcelona, 08193 Barcelona, Spain;
mariateresa.garcia.guasch@uab.cat (M.G.-G.); eduard.escrich@uab.cat (E.E.)
[2] CICECO—Aveiro Institute of Materials, Department of Chemistry, University of Aveiro,
Campus de Santiago, 3810-193 Aveiro, Portugal
* Correspondence: raquel.moral@uab.cat (R.M.); ioladuarte@ua.pt (I.F.D.)

Figures

Figure S1. Heatmaps illustrating the levels of (A) intracellular polar metabolites and (B) lipids in MDA-MB-231 and in MCF-7 cells.

Figure S2. Multivariate analysis of the spectral profiles from MDA-MB-231 cells polar extracts.

Figure S3. Multivariate analysis of the spectral profiles from MCF-7 cells polar extracts.

Tables

Table S1. Metabolite variations in the culture medium of MDA-MB-231 cells treated with HT or LUT, in relation to untreated controls.

Table S2. Metabolite variations in the culture medium of MCF-7 cells treated with HT or LUT, in relation to untreated controls.

Table S3. Metabolite variations in the polar extracts of MDA-MB-231 cells treated with HT or LUT, in relation to untreated controls.

Table S4. Metabolite variations in the polar extracts of MCF-7 cells treated with HT or LUT, in relation to untreated controls.

Figure S1. Heatmaps illustrating the levels of (A) intracellular polar metabolites and (B) lipids in MDA-MB-231 and in MCF-7 cells. Signal integrals were normalied to total area, mean-centered and scaled to Unit Variance.

A

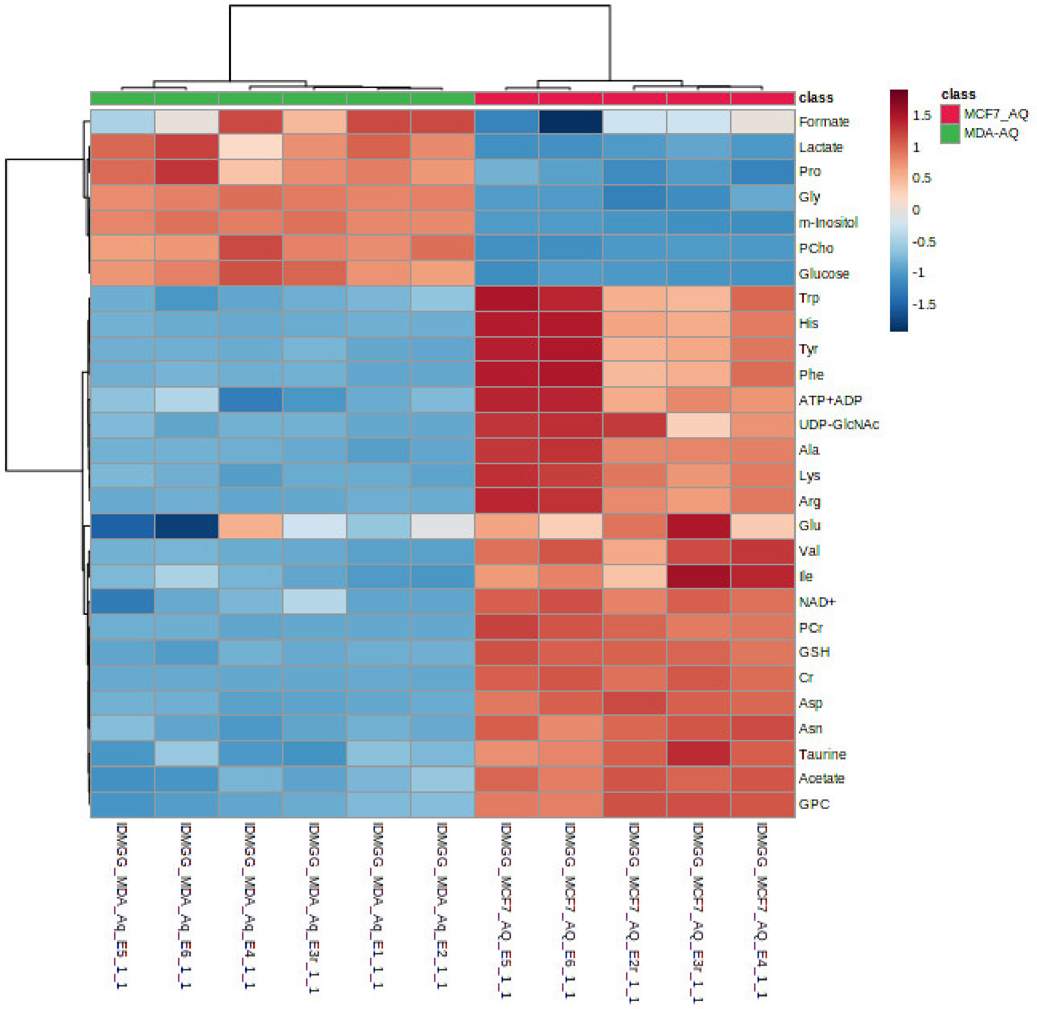

B

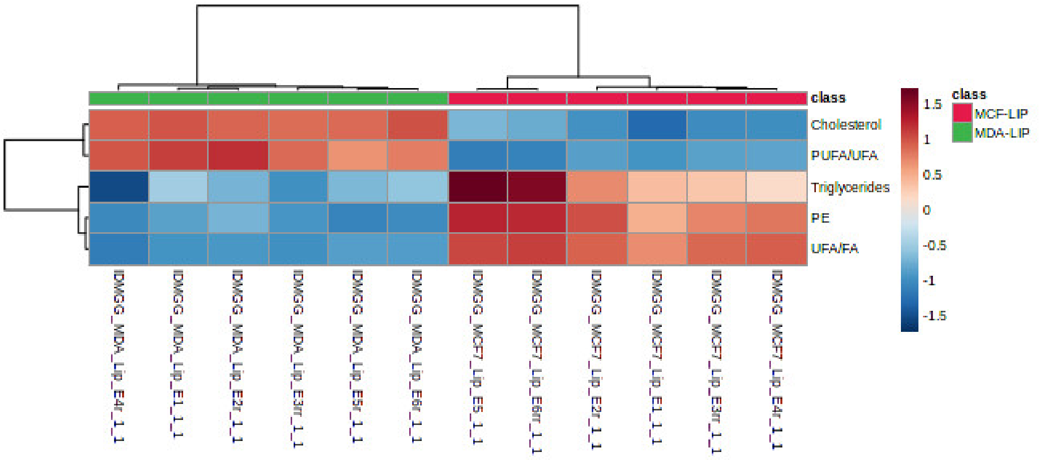

Figure S2. Multivariate analysis of the spectral profiles from MDA-MB-231 cells polar extracts. **(A)** and **(C)** Scores scatter plots obtained by PCA (left) and PLS-DA (right) of cells treated with HT and LUT, respectively. **(B)** and **(D)** PLS-DA LV1 loadings plots colored according to variable importance to the projection (VIP), showing discriminatnt features for HT- and LUT-treated cells, respectively. Three-letter codes used for amino acids; ATP/ADP, adenosine tri/di-phosphate; BCAA, branched-chain amino acids; Cr, creatine; GPC, glycerophosphocholine; GSH, reduced glutathione; m-Ino, myo-inositol.

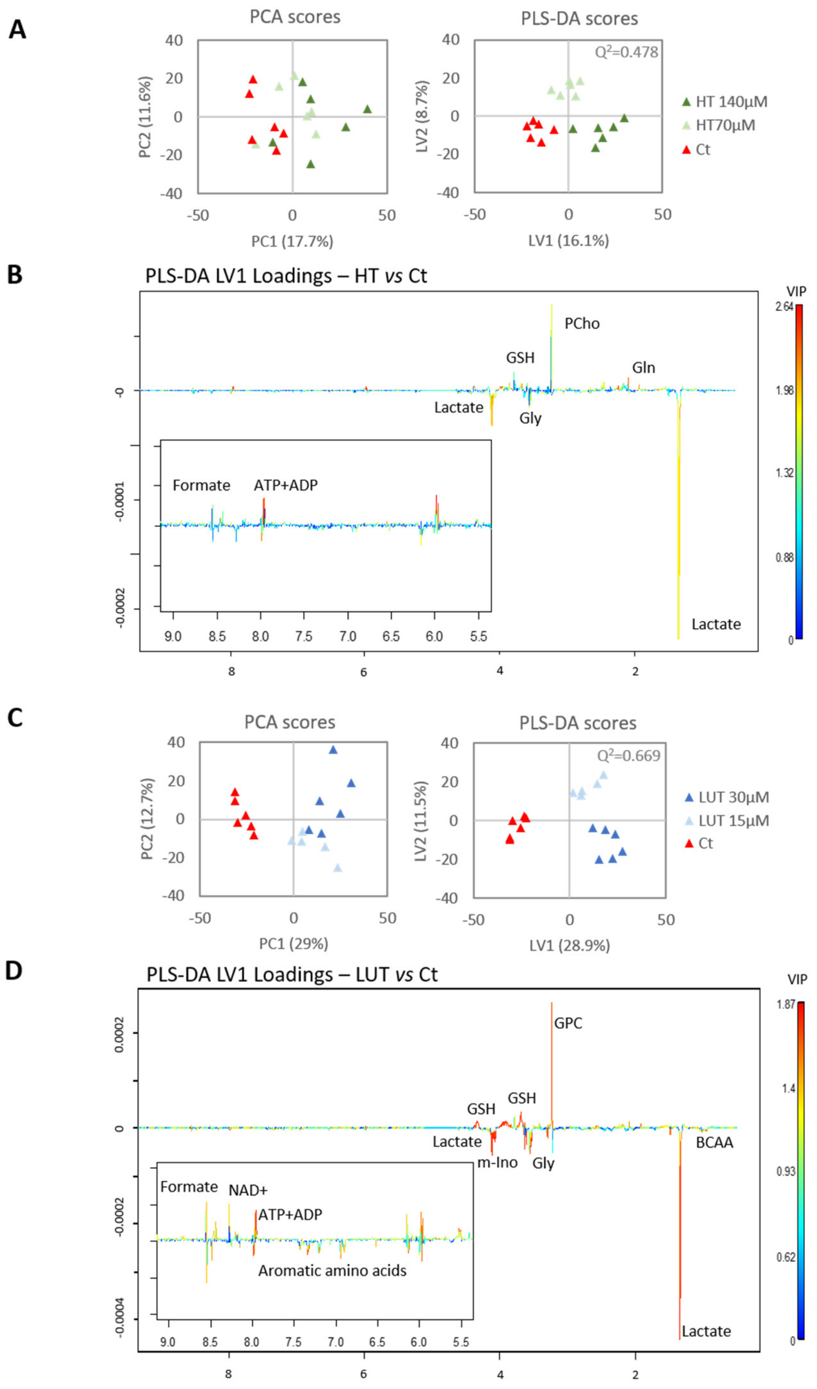

Figure S3. Multivariate analysis of the spectral profiles from MCF-7 cells polar extracts. **(A)** and **(C)** Scores scatter plots obtained by PCA (left) and PLS-DA (right) of cells treated with HT and LUT, respectively. **(B)** and **(D)** PLS-DA LV1 loadings plots colored according to variable importance to the projection (VIP), showing discriminatnt features for HT- and LUT-treated cells, respectively. Three-letter codes used for amino acids; ATP/ADP, adenosine tri/di-phosphate; BCAA, branched-chain amino acids; GSH, reduced glutathione; m-Ino, myo-inositol; NAD^+, nicotinamide adenine dinucleotide; PCho, phosphocholine.

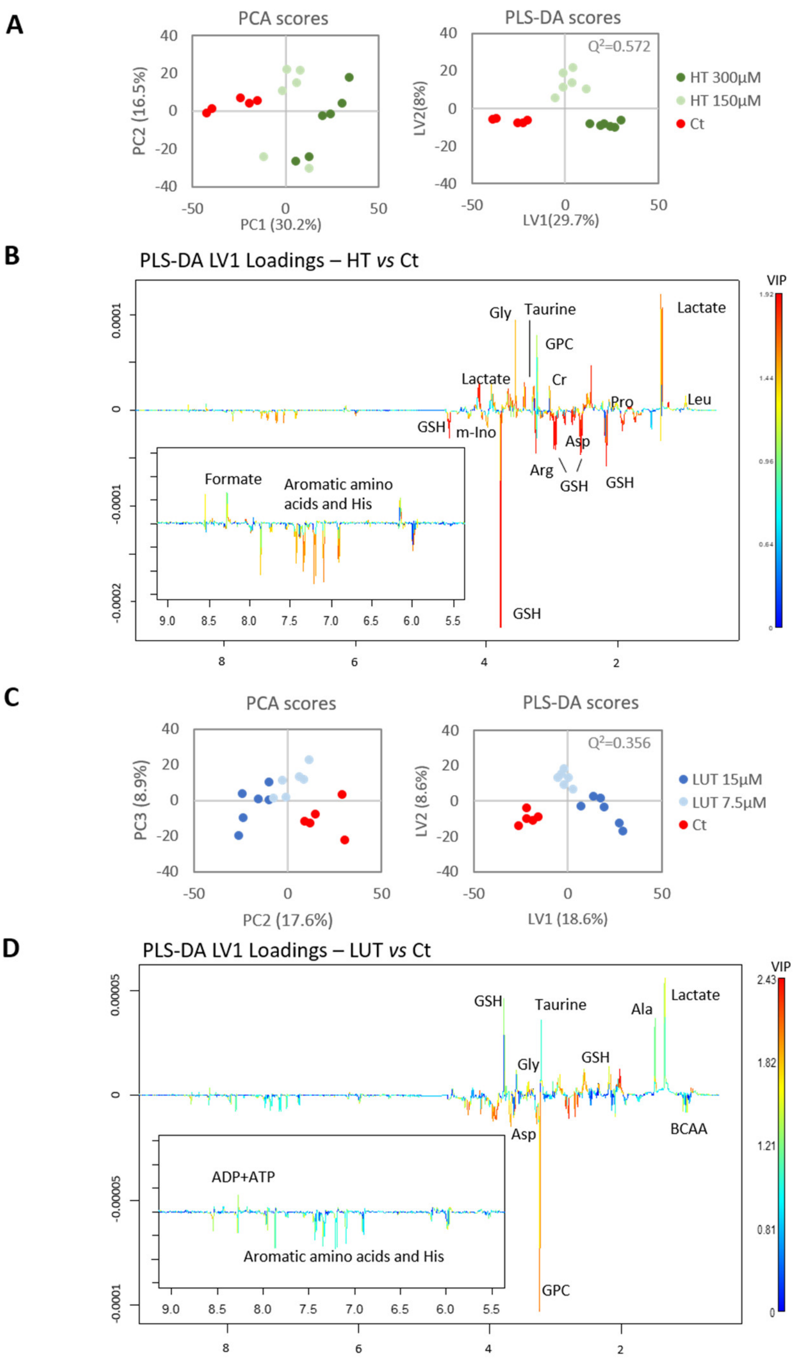

Table S1. Metabolite variations in the culture medium of MDA-MB-231 cells treated with HT or LUT, in relation to untreated controls. The values of % variation are those used to color-code the heatmap shown in Figure 2B.

		HT 70 µM	HT 140 µM	LUT 15 µM	LUT 30 µM
**Acetate**	% Var	0	17.74	16.45	44.35
	±		4.57	3.93	4.03
	ES		2.04	2.21	4.96
	p-value		$7.90x10^{-3}$	$9.72x10^{-3}$	$1.81x10^{-5}$
**Ala**	% Var		-8.87	-9.78	-6.23
	±		3.34	2.92	3.25
	ES		-1.59	-2.01	-1.12
	p-value		$2.50x10^{-2}$	$1.30x10^{-2}$	$8.26x10^{-2}$
**Formate**	% Var	54.17	93.33	31.67	27.78
	±	3.42	6.33	7.68	3.33
	ES	6.51	5.75	2.03	3.96
	p-value	$8.96x10^{-7}$	$2.36x10^{-4}$	$1.76x10^{-2}$	$4.88x10^{-5}$
**Gln**	% Var	0	0	7.77	14.10
	±			3.11	2.94
	ES			1.37	2.45
	p-value			$4.29x10^{-2}$	$1.67x10^{-3}$
**Glu**	% Var	0	0	20.18	0
	±			4.60	
	ES			2.28	
	p-value			$9.04x10^{-3}$	
**Glucose**	% Var	8.65	10.10	23.46	30.59
	±	5.40	4.34	3.88	4.24
	ES	0.85	1.26	3.09	3.57
	p-value	$1.63x10^{-1}$	$7.60x10^{-2}$	$4.56x10^{-3}$	$5.56x10^{-4}$
**Gly**	% Var	11.04	10.58	20.82	23.01
	±	1.58	1.65	1.89	1.62
	ES	3.90	3.47	5.72	7.24
	p-value	$8.96x10^{-4}$	$8.94x10^{-4}$	$9.10x10^{-6}$	$5.03x10^{-6}$
**Glycerol**	% Var	9.62	13.59	-12.05	-26.50
	±	5.06	5.36	4.45	4.80
	ES	0.99	1.36	-1.64	-3.79
	p-value	$1.05x10^{-1}$	$4.58x10^{-2}$	$2.81x10^{-2}$	$7.86x10^{-4}$
**Ile**	% Var	0	0	0	0
	±				
	ES				
	p-value				
**KIC**	% Var	16.67	0	32.38	23.02
	±	5.99		5.25	6.73
	ES	1.46		3.03	1.67
	p-value	$3.71x10^{-2}$		$1.85x10^{-3}$	$1.43x10^{-2}$

		HT 70µM	HT 140µM	LUT 15µM	LUT 30µM
**KIV**	% Var	15.50	16.96	13.45	0
	±	7.84	8.63	7.29	
	ES	1.07	1.04	0.99	
	p-value	1.18x10^{-1}	1.11x10^{-1}	1.52x10^{-1}	
**KMV**	% Var	8.53	8.87	14.11	0
	±	5.34	5.13	4.77	
	ES	0.89	0.95	1.58	
	p-value	1.75x10^{-1}	1.53x10^{-1}	4.19x10^{-2}	
**Lactate**	% Var	11.99	12.42	-7.96	-6.39
	±	2.76	2.91	4.86	3.36
	ES	2.28	2.30	-0.97	-1.08
	p-value	3.32x10^{-3}	3.87x10^{-3}	1.37x10^{-1}	8.04x10^{-2}
**Leu**	% Var	0	0	6.97	7.50
	±			1.63	1.52
	ES			2.36	2.46
	p-value			6.45x10^{-3}	1.76x10^{-3}
**Lys**	% Var	0	0	6.16	7.60
	±			2.68	2.13
	ES		-0.66	1.27	1.85
	p-value			6.34x10^{-2}	7.55x10^{-3}
**Met**	% Var	0	0	11.91	15.52
	±			1.99	1.66
	ES			3.23	4.90
	p-value			4.78x10^{-4}	3.66x10^{-5}
**Pyruvate**	% Var	0	0	0	0
	±				
	ES				
	p-value				
**Ser**	% Var	11.11	18.18	36.80	44.30
	±	4.17	2.81	3.21	3.21
	ES	1.35	3.39	5.53	6.12
	p-value	3.34x10^{-2}	1.72x10^{-3}	1.21x10^{-5}	1.72x10^{-6}
**Val**	% Var	0	0	8.13	8.30
	±			1.71	1.55
	ES			2.61	2.71
	p-value			3.19x10^{-3}	7.83x10^{-4}

Table S2. Metabolite variations in the culture medium of MCF-7 cells treated with HT or LUT, in relation to untreated controls. The values of % variation are those used to color-code the heatmap shown in Figure 2D.

		HT 150µM	HT 300µM	LUT 7.5µM	LUT 15µM
**Acetate**	% Var	90.64	261.10	-5.20	13.88
	±	6.66	9.22	4.42	6.61
	ES	4.99	7.89	-0.64	1.05
	p-value	$6.59x10^{-5}$	$1.49x10^{-4}$	$2.56x10^{-1}$	$8.76x10^{-2}$
**Ala**	% Var	30.97	19.88	11.59	6.48
	±	4.14	2.51	2.85	3.41
	ES	3.45	4.25	2.05	0.98
	p-value	$8.15x10^{-4}$	$3.94x10^{-4}$	$6.70x10^{-3}$	$1.13x10^{-1}$
**Formate**	% Var	52.61	87.11	0	0
	±	10.89	9.40		
	ES	2.04	3.50		
	p-value	$3.84x10^{-3}$	$1.40x10^{-4}$		
**Gln**	% Var	19.63	96.26	-36.60	-32.09
	±	12.88	10.94	21.20	22.94
	ES	0.74	3.22	-1.13	-0.89
	p-value	$2.12x10^{-1}$	$2.61x10^{-4}$	$6.15x10^{-2}$	$1.27x10^{-1}$
**Glu**	% Var	13.67	11.60	11.04	10.64
	±	4.05	4.40	3.69	4.42
	ES	1.69	1.38	1.51	1.22
	p-value	$1.03x10^{-2}$	$3.41x10^{-2}$	$1.99x10^{-2}$	$4.55x10^{-2}$
**Glucose**	% Var	-73.04	-46.62	-55.30	-52.39
	±	17.85	16.07	17.83	17.18
	ES	-3.43	-1.91	-2.28	-2.20
	p-value	$1.14x10^{-3}$	$7.07x10^{-3}$	$2.72x10^{-3}$	$3.69x10^{-3}$
**Gly**	% Var	102.22	217.33	0	0
	±	33.15	11.00		
	ES	1.09	5.19		
	p-value	$8.28x10^{-2}$	$1.16x10^{-5}$		
**His**	% Var	0	-13.22	0	0
	±		2.97		
	ES		-2.64		
	p-value		$1.26x10^{-3}$		
**Ile**	% Var	24.29	41.67	0	0
	±	8.83	9.02		
	ES	1.31	2.04		
	p-value	$4.61x10^{-2}$	$4.67x10^{-3}$		
**KIC**	% Var	0	0	0	0
	±				
	ES				
	p-value				

		HT 150µM	HT 300µM	LUT 7.5µM	LUT 15µM
**KIV**	% Var	-18.41	-37.76	0	0
	±	11.30	12.50		
	ES	-0.96	-1.87		
	p-value	$1.20x10^{-1}$	$9.19x10^{-3}$		
**KMV**	% Var	-13.43	-21.81	0	0
	±	7.73	8.04		
	ES	-0.99	-1.55		
	p-value	$1.06x10^{-1}$	$2.00x10^{-2}$		
**Lactate**	% Var	20.13	8.26	18.07	20.82
	±	3.54	3.33	3.67	3.39
	ES	2.76	1.21	2.41	2.97
	p-value	$7.98x10^{-4}$	$5.63x10^{-2}$	$1.61x10^{-3}$	$7.06x10^{-4}$
**Leu**	% Var	41.48	74.41	0	0
	±	9.48	9.06		
	ES	1.93	3.14		
	p-value	$1.41x10^{-2}$	$6.74x10^{-4}$		
**Lys**	% Var	5.50	5.17	0	-5.28
	±	1.88	1.03		1.89585967
	ES	1.52	2.70		-1.525629128
	p-value	$2.65x10^{-2}$	$9.02x10^{-4}$		0.02509619
**Met**	% Var	-95.15	-95.24	0	0
	±	33.01	30.53		
	ES	-2.93	-2.76		
	p-value	$2.45x10^{-3}$	$2.43x10^{-3}$		
**Phe**	% Var	0	0	0	0
	±				
	ES				
	p-value				
**Pyruvate**	% Var	0	0	0	0
	±				
	ES				
	p-value				
**Thr**	% Var	0	0	0	0
	±				
	ES				
	p-value				
**Tyr**	% Var	0	0	0	0
	±				
	ES				
	p-value				
**Val**	% Var	18.27	31.61	0	0
	±	6.21	6.39		
	ES	1.44	2.24		
	p-value	$3.78x10^{-2}$	$3.24x10^{-3}$		

Table S3. Metabolite variations in the polar extracts of MDA-MB-231 cells treated with HT or LUT, in relation to untreated controls. The values of % variation are those used to color-code the heatmap shown in Figure 4C.

		HT 70µM	HT 140µM	LUT 15µM	LUT 30µM
**Acetate**	% Var	9.38	23.80	45.01	47.73
	±	4.40	5.92	5.15	4.93
	ES	1.07	1.91	4.06	4.43
	p-value	$7.48x10^{-2}$	$5.68x10^{-3}$	$7.99x10^{-5}$	$3.44x10^{-5}$
**ADP+ATP**	% Var	0	0	10.92	14.27
	±			2.61	3.43
	ES			2.22	2.27
	p-value			$3.75x10^{-3}$	$7.11x10^{-3}$
**Ala**	% Var	0	-4.61	-13.55	-18.68
	±			4.00	3.85
	ES			-2.06	-3.01
	p-value			$8.45x10^{-3}$	$8.62x10^{-4}$
**Cr**	% Var	0	-7.25	-8.68	-10.73
	±		3.18	3.43	4.49
	ES		-1.26	-1.40	-1.41
	p-value		$4.52x10^{-2}$	$2.80x10^{-2}$	$3.60x10^{-2}$
**Formate**	% Var	13.11	25.41	48.52	45.57
	±	5.64	6.01	5.86	6.23
	ES	1.11	2.00	3.61	3.27
	p-value	$7.30x10^{-2}$	$5.25x10^{-3}$	$9.59x10^{-5}$	$1.92x10^{-4}$
**Gln**	% Var	9.98	10.61	0	-12.92
	±	3.59	3.44		3.78
	ES	1.43	1.56		-1.93
	p-value	$2.61x10^{-2}$	$1.64x10^{-2}$		$6.01x10^{-3}$
**Gly**	% Var	3.65	-1.92	-7.61	-22.97
	±			1.91	2.98
	ES			-2.38	-5.11
	p-value			$4.61x10^{-3}$	$2.87x10^{-4}$
**GPC**	% Var	0	8.45	38.17	78.82
	±		3.81	4.92	7.07
	ES		1.14	3.89	4.96
	p-value		$6.08x10^{-2}$	$7.30x10^{-4}$	$6.87x10^{-4}$
**GSH**	% Var	13.67	8.31	19.04	16.93
	±	4.82	5.20	3.92	5.17
	ES	1.48	0.82	2.38	1.71
	p-value	$2.65x10^{-2}$	$1.57x10^{-1}$	$1.74x10^{-3}$	$1.59x10^{-2}$
**Ile**	% Var	4.43	0	-16.77	-11.38
	±			1.92	2.10
	ES			-4.83	-3.01
	p-value			$6.40x10^{-5}$	$3.97x10^{-4}$

		HT 70µM	HT 140µM	LUT 15µM	LUT 30µM
**Lactate**	% Var	-4.98	-15.73	-36.08	-34.29
	±		4.82	4.75	5.04
	ES		-1.89	-4.69	-4.24
	p-value		$6.20x10^{-3}$	$4.12x10^{-5}$	$3.77x10^{-5}$
**Leu**	% Var	3.54	0	-8.54	-6.26
	±			1.75	1.67
	ES			-2.66	-2.00
	p-value			$9.80x10^{-4}$	$5.67x10^{-3}$
**m-Ino**	% Var	2.48	-3.23	-17.11	-21.30
	±			2.63	3.17
	ES			-4.08	-4.38
	p-value			$2.71x10^{-4}$	$3.67x10^{-4}$
**NAD+**	% Var	0	14.74	28.00	30.11
	±		7.28	7.16	4.53
	ES		1.01	2.00	3.10
	p-value		$9.36x10^{-2}$	$1.08x10^{-2}$	$3.06x10^{-4}$
**Pcho**	% Var	10.37	11.15	0	-13.06
	±	3.81	4.13		5.83
	ES	1.36	1.36		-1.33
	p-value	$3.29x10^{-2}$	$3.02x10^{-2}$		$4.34x10^{-2}$
**PCr**	% Var	3.31	-3.89	-4.54	-9.94
	±				2.52
	ES				-2.44
	p-value				$7.63x10^{-3}$
**Phe**	% Var	0	-4.06	-13.59	-16.15
	±			2.95	3.57
	ES			-2.76	-2.81
	p-value			$1.15x10^{-3}$	$1.97x10^{-3}$
**Taurine**	% Var	6.24	4.73	13.46	20.49
	±	3.69		3.95	4.11
	ES	0.97		1.91	2.72
	p-value	$1.56x10^{-1}$		$2.25x10^{-2}$	$5.96x10^{-3}$
**Tyr**	% Var	4.17	0	-10.89	-11.40
	±			3.17	2.92
	ES			-1.95	-2.18
	p-value			$5.67x10^{-3}$	$3.03x10^{-3}$
**UDP-GlcNAC**	% Var	0	14.72	28.15	46.04
	±		2.30	3.59	4.59
	ES		3.18	4.20	5.04
	p-value		$5.45x10^{-4}$	$1.36x10^{-3}$	$8.46x10^{-4}$
**Val**	% Var	6.22	3.06	-15.53	-6.43
	±	2.42		1.85	1.91
	ES	1.43		-4.81	-1.89
	p-value	$4.00x10^{-2}$		$1.02x10^{-5}$	$7.00x10^{-3}$

Table S4. Metabolite variations in the polar extracts of MCF-7 cells treated with HT or LUT, in relation to untreated controls. The values of % variation are those used to color-code the heatmap shown in Figure 4D.

		HT 150µM	HT 300µM	LUT 7.5µM	LUT 15µM
**ADP+ATP**	% Var	13.04	17.85	5.44	0
	±	5.83	4.39	3.03	
	ES	1.07	1.92	0.95	
	p-value	8.03×10^{-2}	7.36×10^{-3}	1.15×10^{-1}	
**Ala**	% Var	21.10	-12.90	25.14	21.34
	±	4.66	4.93	4.45	4.42
	ES	2.27	-1.63	2.80	2.49
	p-value	3.46×10^{-3}	2.91×10^{-2}	1.15×10^{-3}	3.18×10^{-3}
**Arg**	% Var	-21.98	-48.50	-7.92	-11.58
	±	1.41	3.10	2.36	2.55
	ES	-9.91	-11.00	-1.81	-2.75
	p-value	8.83×10^{-8}	1.23×10^{-7}	9.72×10^{-3}	3.77×10^{-3}
**Asn**	% Var	52.59	42.59	14.81	28.44
	±	3.76	2.87	6.88	5.85
	ES	5.89	7.10	1.03	2.43
	p-value	1.79×10^{-6}	3.19×10^{-5}	8.62×10^{-2}	4.92×10^{-3}
**Asp**	% Var	-41.37	-63.80	-22.63	-36.29
	±	3.36	3.19	3.12	3.50
	ES	-8.63	-17.57	-4.47	-7.24
	p-value	6.91×10^{-8}	1.09×10^{-8}	1.71×10^{-5}	2.35×10^{-6}
**Cr**	% Var	22.59	33.00	0	0
	±	5.60	6.06		
	ES	1.85	2.36		
	p-value	9.03×10^{-3}	3.02×10^{-3}		
**Formate**	% Var	21.71	52.41	0	0
	±	11.04	12.64		
	ES	0.99	1.72		
	p-value	1.16×10^{-1}	1.09×10^{-2}		
**Gln**	% Var	37.66	26.95	-6.23	-15.47
	±	11.93	2.88	4.39	5.45
	ES	1.32	4.81	-0.81	-1.76
	p-value	4.33×10^{-2}	2.78×10^{-4}	1.76×10^{-1}	1.66×10^{-2}
**Gly**	% Var	134.15	138.47	0	0
	±	17.54	7.41		
	ES	2.24	5.66		
	p-value	5.19×10^{-3}	3.09×10^{-6}		
**GPC**	% Var	-8.42	7.59	-7.95	-16.13
	±	2.92	1.93	2.40	2.08
	ES	-1.60	2.11	-1.88	-4.83
	p-value	1.57×10^{-2}	5.17×10^{-3}	7.17×10^{-3}	5.25×10^{-5}

		HT 150uM	HT 300uM	LUT 7.5uM	LUT 15uM
**GSH**	% Var	-39.93	-54.17	10	11.08
	±	3.00	3.53	1.42	2.17
	ES	-8.92	-11.36	3.79	2.76
	p-value	1.60×10^{-7}	3.87×10^{-8}	1.80×10^{-4}	2.18×10^{-3}
**His**	% Var	-9.90	-44.00	-15.16	-15.84
	±	4.29	4.47	4.95	4.11
	ES	-1.39	-7.86	-1.84	-2.39
	p-value	4.29×10^{-2}	1.28×10^{-4}	9.15×10^{-3}	5.52×10^{-3}
**Ile**	% Var	15.20	0	-13.64	-11.00
	±	7.27		4.97	5.26
	ES	1.00		-1.66	-1.26
	p-value	9.31×10^{-2}		1.73×10^{-2}	5.80×10^{-2}
**Lactate**	% Var	44.18	66.72	0	0
	±	9.11	6.75		
	ES	2.07	3.97		
	p-value	4.00×10^{-2}	5.03×10^{-5}		
**Leu**	% Var	36.22	43.68	-11.65	-5.82
	±	1.24	3.44	2.45	2.01
	ES	12.79	5.17	-2.59	-1.70
	p-value	$7.18E \times 10^{-9}$	1.01×10^{-4}	2.00×10^{-3}	2.73×10^{-2}
**Lys**	% Var	-17.17	-36.18	0	0
	±	5.09	5.62		
	ES	-2.19	-4.75		
	p-value	1.06×10^{-2}	3.56×10^{-4}		
**m-Ino**	% Var	-34.03	-43.24	-17.12	-17.57
	±	7.59	8.07	7.99	7.68
	ES	-3.28	-4.18	-1.33	-1.43
	p-value	2.56×10^{-3}	8.67×10^{-4}	4.65×10^{-2}	4.03×10^{-2}
**NAD+**	% Var	12.19	0	15.42	8.72
	±	3.11		3.07	2.01
	ES	1.89		2.37	2.37
	p-value	8.88×10^{-3}		2.87×10^{-3}	3.81×10^{-3}
**Pcho**	% Var	0	48.66	-21.77	0
	±		4.68	1.95	
	ES		4.15	-7.09	
	p-value		2.68×10^{-4}	9.93×10^{-7}	
**PCr**	% Var	0	0	9.50	5.30
	±			1.73	2.31
	ES			3.01	1.28
	p-value			1.29×10^{-3}	5.67×10^{-2}
**Phe**	% Var	-6.71	-30.93	-16.47	-17.91
	±	4.42	4.31	4.34	4.21
	ES	-0.90	-5.26	-2.44	-2.67
	p-value	1.57×10^{-1}	7.83×10^{-4}	6.04×10^{-3}	4.62×10^{-3}

		HT 150uM	HT 300uM	LUT 7.5uM	LUT 15uM
**Pro**	% Var	29.95	20.41	14.05	28.24
	±	5.21	1.93	2.36	1.47
	ES	2.50	5.21	2.94	9.60
	p-value	3.10×10^{-3}	5.69×10^{-6}	4.18×10^{-4}	3.17×10^{-6}
**PyroGlu**	% Var	38.76	68.33	0	0
	±	7.00	5.68		
	ES	2.60	5.12		
	p-value	2.19×10^{-3}	1.19×10^{-4}		
**Pyruvate**	% Var	23.61	12.50	0	0
	±	4.36	3.73		
	ES	2.59	1.77		
	p-value	9.91×10^{-4}	1.42×10^{-2}		
**Taurine**	% Var	18.81	36.18	6.00	8.49
	±	2.21	1.96	1.72	1.59
	ES	4.08	8.19	1.89	2.92
	p-value	4.58×10^{-5}	1.61×10^{-7}	8.75×10^{-3}	1.19×10^{-3}
**Thr**	% Var	0	-13.67	9.58	11.40
	±		4.40	4.81	5.35
	ES		-1.88	1.02	1.15
	p-value		9.54×10^{-3}	9.11×10^{-2}	8.41×10^{-2}
**Trp**	% Var	0	-36.41	-13.59	-17.50
	±		5.20	5.57	5.20
	ES		-5.28	-1.48	-2.11
	p-value		5.33×10^{-4}	2.97×10^{-2}	8.49×10^{-3}
**Tyr**	% Var	-12.77	-28.66	0	0
	±	8.92	10.20		
	ES	-0.92	-1.95		
	p-value	1.83×10^{-1}	1.66×10^{-2}		
**Val**	% Var	11.60	0	-14.25	-14.12
	±	6.70		3.99	3.98
	ES	0.84		-2.21	-2.18
	p-value	1.47×10^{-1}		5.76×10^{-3}	6.47×10^{-3}
